# Molecular Insights Into the Causes of Human Thymic Hypoplasia With Animal Models

**DOI:** 10.3389/fimmu.2020.00830

**Published:** 2020-05-05

**Authors:** Pratibha Bhalla, Christian A. Wysocki, Nicolai S. C. van Oers

**Affiliations:** ^1^Department of Immunology, The University of Texas Southwestern Medical Center, Dallas, TX, United States; ^2^Department of Pediatrics, The University of Texas Southwestern Medical Center, Dallas, TX, United States; ^3^Department of Microbiology, The University of Texas Southwestern Medical Center, Dallas, TX, United States

**Keywords:** thymus development, thymic hypoplasia, TECs, mesenchymal cells, 22q11.2 deletion syndrome, *PAX1*, *FOXN1*, *CHD7*

## Abstract

22q11.2 deletion syndrome (DiGeorge), CHARGE syndrome, Nude/SCID and otofaciocervical syndrome type 2 (OTFCS2) are distinct clinical conditions in humans that can result in hypoplasia and occasionally, aplasia of the thymus. Thymic hypoplasia/aplasia is first suggested by absence or significantly reduced numbers of recent thymic emigrants, revealed in standard-of-care newborn screens for T cell receptor excision circles (TRECs). Subsequent clinical assessments will often indicate whether genetic mutations are causal to the low T cell output from the thymus. However, the molecular mechanisms leading to the thymic hypoplasia/aplasia in diverse human syndromes are not fully understood, partly because the problems of the thymus originate during embryogenesis. Rodent and Zebrafish models of these clinical syndromes have been used to better define the underlying basis of the clinical presentations. Results from these animal models are uncovering contributions of different cell types in the specification, differentiation, and expansion of the thymus. Cell populations such as epithelial cells, mesenchymal cells, endothelial cells, and thymocytes are variably affected depending on the human syndrome responsible for the thymic hypoplasia. In the current review, findings from the diverse animal models will be described in relation to the clinical phenotypes. Importantly, these results are suggesting new strategies for regenerating thymic tissue in patients with distinct congenital disorders.

## Introduction

Thymic hypoplasia is a common transient condition seen in newborns, particularly for premature babies (1, 2). A short-lived hypoplasia of the thymus can occur at any age due to infections, diverse forms of stress, pregnancy, alcoholism, malnutrition, and radiation exposure (3–5). In the elderly, a severe and everlasting involution of the thymic tissue is a well-recognized consequence of the aging process (6, 7). There are several genetic disorders in humans that result in permanent hypoplasia or occasional aplasia of the thymus evident at birth. These genetic disorders often lead to severe combined immunodeficiency (SCID) (8). The mutations can be monogenic or multigenic, impacting either the patterning of the thymic anlage, the thymic stromal cell populations, and/or the developing thymocytes. The stromal cell populations include mesenchymal cells, TECs and endothelial cells. Clinical conditions known to impact these stromal cell populations are chromosome 22q11.2 deletion syndrome (22q11.2del), also referred to as DiGeorge syndrome, Coloboma-heart defects-atresia choanae-retardation of growth-genital abnormalities-ear (CHARGE) syndrome arising from mutations in *Chromodomain Helicase DNA Binding Protein 7* (*CHD7*), Nude/SCID due to autosomal recessive mutations in *Forkhead Box N1* (*FOXN1*), otofaciocervical syndrome type 2 (OTFCS2) due to mutations in *PAX1*, as well as mutations in *TBX1* (located within the chromosome 22q11.2 locus) and *TBX2* (Table 1) (9–17). Hypoplasia/aplasia of the thymus can also arise in a developing fetus via teratogen exposures; diabetic- or retinoic acid- induced embryopathies (18–21). In the current review, the genetic mutations that affect the stromal cell populations needed for the formation and/or function of the thymus are described. Since these mutations often influence the specification of the thymus during embryogenesis, detailed mechanistic insights have come from mouse, rat and even zebrafish models.

**Table 1:** Stromal cell intrinsic causes of thymic hypoplasia (T^−/lo^B^+^NK^+^) from specific human clinical disorders.

**Disease name**	**Frequency in the population**	**Genes Affected (# SNPs in ClinVar database^a^)**	**Thymic hypoplasia (% of patients)**	**Mouse models**	**Stromal cell populations affected**
22q11.2 deletion syndrome	1 in 4000	>105 genes [46 coding, 7 miRNAs, 12 lncRNAs, 2 snoRNAs, rest are pseudogenes]	60–70% <1% with aplasia of the thymus	Chromosome 16 ortholog deletions, *Tbx1* targeted mice	Stromal cells (mesenchymal, endothelial, epithelial)
CHARGE syndrome	1 in 8500-10,000	*CHD7* (SNPs = 973)	50%	*Chd7* knockout and knock-in lines	Neural crest cells (mesenchyme)-TECs
Nude/SCID^b^ and SCID	Rare	*FOXN1* (SNPs = 126)	90%	*Foxn1* knockout and knock-in lines	cTECs and mTECs
Otofaciocervical syndrome	Rare	*PAX1* (SNPS = 29)	100%	*Undulated* series and *Pax1* knockout lines	Endodermally-derived epithelial cells
22q11.2-like cardiovascular and skeletal disorder	Rare	*TBX2* (SNPs = 25)	100%	*Tbx2* knockout mice	Stromal cells
Maternal diabetes	3–9% of pregnancies	Multiple genes e.g., *CybpA1*	18% of those needing thymic transplant	Gestational and pre-gestational diabetes	Stromal (mesenchymal, epithelial, endothelial)
Fetal retinoid syndrome	5–20% malformation rates in live births	Multiple genes e.g., *Tbx*1, *Tbx2, Bmp4, Foxn1*	Unknown	Retinoic acid injections Enzyme KO mice	Stromal (mesenchymal, epithelial, endothelial)

a *single nucleotide polymorphisms = SNPs, reported in ClinVar database*.

b *severe combined immunodeficiency = SCID*.

## Review Article

### Overview of Murine Thymus Development During Embryogenesis

**Figure 1:**
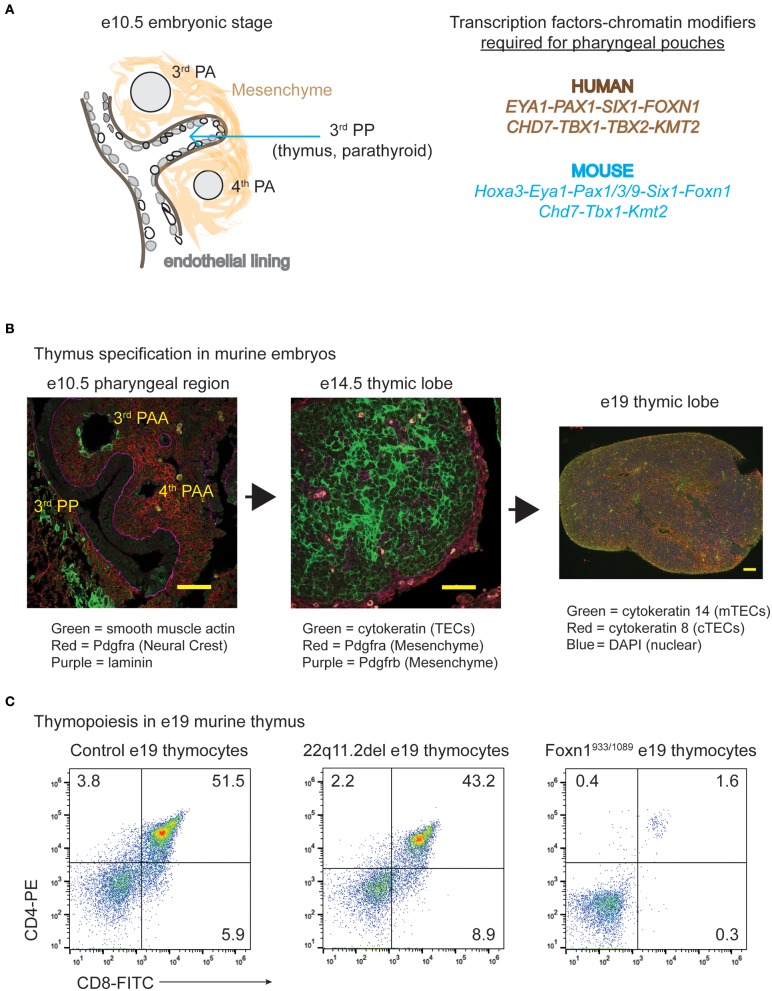
The specification and expansion of the thymus during embryogenesis in normal and disease states. **(A)** Cartoon diagram illustrating the development process of the thymus along with the various transcription factors and gene products required. The genes that have roles in the specification of the human pharyngeal apparatus that affects the 3rd pharyngeal pouch (thymus and parathyroid) are shown in brown, while those confirmed importance for these processes in mice are in blue. **(B)** Transverse tissue sections or intact thymic lobes were isolated from normal embryos at the indicated ages of gestation. The transverse sections or whole mounts of the tissue were prepared for immunohistochemistry and H&E staining. Antibodies against vascular smooth muscle, pdgfr-a (alpha) marking the mesenchymal cells and thymic capsule, pdgfr-b (beta) delineating mesenchymal cells and the vasculature, cytokeratin (TECs) and laminin were used, with the colors indicated below the image. **(C)** Thymocyte subset distributions present in e19–19.5 embryonic thymuses from control C57BL/6 mice, those modeling 22q11.2 deletion syndrome (Tbx1^neo2/neo2^) and those with compound heterozygous mutations in *Foxn1* (Foxn1^933/1089^) are shown. The *Foxn1* mutations genocopy that identified in a human patient (22). Both control and 22q11.2del thymuses have similar distributions of CD4 and CD8 thymocyte subset percentages, suggesting normal TEC functions. The *Foxn1* mutant mice are blocked at the CD4^−^CD8^−^ subset, indicating a severe TEC dysfunction.

The thymus and parathyroid glands develop from the 3rd pharyngeal pouch (PP), a temporary embryonic structure that begins as an evagination of endothelial cells from the gut tube between e9.5–10.5 (Figures 1A,B) (23). The formation of the 3rd PP requires several transcription factors including *Paired box gene* family members, *Sin oculus homolog 1* (*Six1*), and *Eyes absent 1* (*Eya1*) (23–25). As the 3rd PP forms, an endothelial layer within this region is surrounded by an area of neural crest-derived mesenchymal cells. Ectodermal in origin, these mesenchymal cells secrete bone morphogenic protein 4 (bmp4) and bone morphogenic protein 2 (bmp2) to support the patterning of the 3rd PP (26). The targeted deletion of *bmp4* in neural crest cells leads to a reduced contraction of the mesenchymal cells in the 3rd PP (26). This results in morphogenesis defects of both the thymus and parathyroid domains, which are delineated by the expression of *Forkhead box n1* (*Foxn1*) and *Glia cells missing 2* (*Gcm2*), respectively. The demarcation of the thymus domain by *bmp4* is balanced by *Sonic hedgehog* (Shh), which establishes the dorsal parathyroid region (27). Interestingly, the initial specification of the thymus and parathyroid regions can occur in the absence of neural crest cells, which are lacking in splotch mutant mice, which have mutations in the *Paired box gene 3* (*Pax3*) transcription factor (28). *Paired box gene 1* (*Pax1*) is a related family member also involved in the development of the thymic anlage (15, 29). A *Pax1* deficiency in mice leads to mild hypoplasia of the thymus (29, 30). Interestingly, *PAX1* autosomal recessive mutations in humans leads to a more severe hypoplasia of the thymus (13, 15, 17).

With regards to the stromal cell populations, the neural crest-derived mesenchymal cells have at least three distinct roles in the development of the thymic tissue. First, these cells form the thymic capsule and vasculature, establishing the overall structure of the thymus. Noteworthy, the mechanical removal of the mesenchymal capsule using e12.5 fetal thymic lobes renders the tissue hypoplastic (31–33). Yet, the development and proportions of thymocytes subsets are normal in these mesenchymal-depleted hypoplastic tissues, revealing intact TEC functions in the setting of their reduced numbers. Second, the mesenchymal cells enable the expanding thymic lobes to detach from the pharynx between e11.5–12.5, with each lobe from the right and left 3rd PP pairing and descending into the mediastinum. This process requires both *Pax3* and *Homeobox a3 (Hoxa3*) transcription factors, with the targeted deletion of *Hoxa3* in neural crest cells resulting in smaller sized thymic lobes remaining attached to the pharynx (28, 34). Third, the mesenchymal cells support thymic epithelial cell (TECs) expansion and differentiation. This involves a combination of ligands and growth factors produced by mesenchymal cells; bmp4, bmp2, fibroblast and insulin growth factors, wnt proteins, and retinoic acid (32, 33, 35–38). Cross-talk between the mesenchymal cells and TECs facilitates thymic tissue expansion, differentiation of TECs into cortical and medullary subsets and recruitment of hematopoietic thymic seeding progenitors (39, 40). The hematopoietic progenitors arrive in timed waves, with the first cells appearing prior to the vascularization of the thymic tissue (41, 42). Following tissue vascularization and remodeling of the epithelia into a 3-dimensional meshwork, the thymic seeding progenitors enter through the cortical-medullary junction (41). These progenitor cells, through a process of cell-cell interactions with TECs, develop into thymocytes. Consistent with the theme of cross-communication among the various cell types in the thymus, ligands expressed by the thymocytes further support the differentiation and expansion of TECs. For example, immature thymocytes are needed for the proper expansion of cTECs during late stages of embryogenesis (43). The cortical TECs positively select T cells expressing the correct T cell receptor (Tcr) specificity for self-peptides embedded by major histocompatibility molecules (44–46). In addition, the emergence of mature SP thymocytes enhances mTEC differentiation and proliferation by releasing epidermal growth factor (Egf) and lymphotoxin and expressing CD40L and RANKL (47–49). The mTECs ensure deletion of potentially autoreactive T cells and enable T regulatory cell selection (44–46). Of note, there are some distinctions between mouse and human thymic tissue specification during embryogenesis (25). Differing contributions of *Pax1* and *Pax9* is one such example, as detailed in the section on otofaciocervical syndrome type 2 (17). In addition, unlike mice, both humans and rats express MHC class II on developing thymocytes and these cells can support the selection and maturation of CD4 single positive cells (50–52). Several articles in the current series “new insights into thymic functions during stress, aging, and in disease settings” as well as other reviews have provided detailed information about the development and contribution of TECs in thymopoiesis (53, 54). The current review will focus on the TECs and other stromal cell types affected by selected clinical disorders.

### 22q11.2 Deletion Syndrome (DiGeorge Syndrome)

Chromosome 22q11.2 deletion syndrome (22q11.2del; OMIM #188400) is a common human disorder (frequency of 1/4000), resulting in variable and complex congenital malformations (8, 55–58). The congenital defects can include thymic hypoplasia, outflow track problems of the heart, hypoparathyroidism, dysmorphic facial features, and/or other midline organ involvement (Table 1). Additional complications for children with 22q11.2del include developmental delay, and over time, neurological problems such as schizophrenia and autism, malignancy, and/or autoimmunity (57–61). Most individuals with 22q11.2del have a 3 Mb microdeletion on chromosome 22, resulting in a hemizygosity of nearly 106 genes (8, 58, 60, 62, 63). A smaller, nested deletion of 1.5 Mb creates a haploinsufficiency of 30 genes, which occurs in 5–8% of 22q11.2del patients (8, 58, 60).

Thymic hypoplasia is reported for 60–70% of individuals with 22q11.2del (56, 58, 64). Due to their thymic hypoplasia, 22q11.2del patients have an average 5-fold reduction in the number of T cell receptor excision circles (TRECs) compared to matched controls, with TRECs measuring the circulating naïve T cells emerging from the thymus (56, 65, 66). In rare cases a patient with 22q11.2del may have complete thymic aplasia, resulting in near-complete absence of autologous T cells, defined by <50 naïve CD3^+^ T cells per microliter of peripheral blood (14). An effective clinical treatment option for such a patient is an allogeneic thymic tissue transplant, first depleted of thymocytes prior to the placement of small fragments of this tissue within the quadricep muscles (67–70). The thymic stromal tissue consists of TECs, mesenchymal cells, and endothelial cells (71). Upon transplant, the stromal tissue recruits host-derived hematopoietic cells that mature into thymocytes (70, 72). A remarkable feature of this thymus transplantation procedure is the successful selection of TCR-expressing T cells recognizing peptides presented by host (recipient) antigen-presenting cells (12, 70, 72, 73). However, the processes of both positive and negative selection and that of MHC restriction of the developing T cells are not completely understood in these thymic tissue transplants. The positive selection of host T cells in a donor thymus MHC (HLA) background could be caused by recipient-derived epithelial progenitor cells (74). Alternatively, the developing human thymocytes could promote positive selection as these cells express MHC class II molecules (50). When MHC class II is forcibly expressed on murine thymocytes, such cells can now positively select CD4 T cells (51, 52). The thymocyte-selected CD4 single positive cells formed in these mouse models are different than conventional CD4 T cells (75). They express the promyelocytic leukemia zinc finger protein (plzf) and produce both gamma-interferon and IL-4, reflecting more innate-like responses (75). The thymic transplants for 22q11.2del patients can additionally enable T regulatory cell development (69, 76). In normal thymopoiesis, these Tregs develop through interactions with medullary TECs. Negative selection is similarly not well-understood following thymus tissue grafting, with the developing T cells tolerant to both the donor and host MHC (76). It is likely that host dendritic cells along with donor mTECs tolerizing/eliminating any developing T cells targeting either host and donor peptide-MHC complexes (53, 68, 76).

Not all 22q11.2del patients who have a severe hypoplasia of the thymus are grafted with an allogeneic thymus (77, 78). Thus, matched sibling and sometimes unrelated bone marrow transplants have been successfully used to treat 22q11.2del patients who have a severe thymic hypoplasia (limited TRECs) (77–81). The recipient 22q11.2del patients have normal T cell functions and humoral immunity, suggesting T cell reconstitution. However, the majority of the donor T cells have a memory phenotype and a limited TCR repertoire (77, 78). In the short term, there is no difference reported in the mortality for the patients receiving a thymic tissue vs. those with a bone marrow transplant (80, 81). This conclusion will require a long-term longitudinal study comparing infection and survival rates with a larger cohort. However, the lack of naïve T cell development in the bone marrow recipients is of clinical concern for 22q11.2del patients and as described in subsequent sections, individuals with *FOXN1* and *PAX1* mutations (82).

An important take-home message from the clinical approaches to treat 22q11.2del patients is that the deletion primarily impacts the stromal cells of the thymus. Yet, which stromal cell type(s) is affected by 22q11.2del remains unknown. One group has analyzed thymuses isolated from 22q11.2del patients, available since this tissue is often removed to allow surgical access to the heart (83). The most distinguishing feature of the thymuses from 22q11.2del patients is its smaller size compared to age-matched control tissues (83). Thymopoiesis appears normal, as the percentage of CD4^−^CD8^−^ (DN), CD4^+^CD8^+^ (DP), and CD4^+^CD8^−^, and CD4^−^CD8^+^ (SP) thymocyte subsets in the hypoplastic tissues is similar to that seen with control samples. The medullary region does appear smaller in the 22q11.2del samples, although the levels of a key gene expressed in medullary TECs, *Autoimmune regulator* (*AIRE)*, is not statistically different from controls (83). Yet, the number of thymic CD4^+^Foxp3^+^ T regulatory cells (Tregs) is diminished in the hypoplastic lobes and these cells have less suppressive capabilities compared to controls (83). It remains unknown why this difference exists but may explain the higher prevalence of autoimmune cytopenias in the 22q11.2del cohort (56, 84, 85). The number of these CD4^+^ Tregs is also decreased in peripheral tissues, but this arises from the generalized T cell lymphopenia affecting most T cell subsets in the setting of 22q11.2del (11, 85–89).

The congenital hypoplasia/aplasia of the thymus caused by 22q11.2del occurs during the patterning of the pharyngeal apparatus in embryos (58, 90–92). This is best revealed in mice, as comparative analyses between normal embryos and those obtained from 22q11.2del mouse models suggest patterning defects of the pharyngeal pouches and arches (61, 90, 91, 93–95). The 22q11.2del mouse lines were initially developed with orthologous deletions on murine chromosome 16 to identify genes causal to the congenital malformations (Table 2). This led to the realization that the principal cause of the congenital defects was linked to a haploinsufficiency of the *T-box Transcription Factor 1* (*TBX1*) (90, 91, 93, 94, 96). TBX1 interacts with members of the Histone-lysine N-methyltransferase (KMT2)-family, activating the low level transcription of over 2,000 genes (97). Interestingly, the penetrance and severity of the congenital malformations due to a haploinsufficiency of *TBX1* varies considerably in the mouse models, which recapitulates the wide range of differences among individual 22q11.2del patients (Table 2). Emerging evidence suggests this variability is due to a combination of genetic and epigenetic regulators, both within and outside chromosome 22q11.2, which influence all the clinical phenotypes of 22q11.2del (8, 98, 99).

**Table 2:** Mouse models of human clinical disorders leading to hypoplasia or aplasia of the thymus.

**Genetic mutation**	**Impact on the thymus**	**Alopecia/nail cornification**	**Stage of developmental block in thymopoiesis**	**Mechanistic insights**
**Mouse models of 22q11.2 deletion syndrome**				
LgDel	Rare mild hypoplasia	None	None	30 genes, including *Tbx1* are haploinsufficient causing partially penetrant cardiac anomalies, minimal effect on thymus
Df (16)A	Rare mild hypoplasia	None	None	
Df1/+	Rare mild hypoplasia	None	None	
Tbx1^−/−^	Aplasia	None	DN1^a^	Early embryonic lethal
Tbx1^neo2/+^ and Tbx1^neo/+^	Mild hypoplasia	None	None	50 and 70% normal *Tbx1* levels, respectively, enabling a gene dosage analysis and showing Tbx1 key to congenital abnormalities
Tbx1^+/−^	Mild hypoplasia	None	None	*Tbx1* haploinsufficient (see above with Df and Lg series)
Tbx1^neo2/neo2^	Severe hypoplasia	None	None	35% normal *Tbx1* levels leads to more penetrant and severe congenital malformations
**Mouse models of CHARGE syndrome:** ***Chd7*** **mutations created**				
*Chd7* point mutations	Not reported	None	None	Affects the cardiac tissue and malformations of the ear canal
*Chd 7* ENU^b^ mutations	Not reported	None	None	12 distinct mutations matching human mutations. Phenotypes mimic human disorder
*Chd7* gene trap	Not reported	None	None	Three mutations (exons 1, 4, and 34) with effects as with ENU
*Chd7* gene knockout		None	None	Exon 2 targeted, clinical phenotypes as with humans
*Chd7*^+/xk^ gene trap	Hypoplasia	None	None	11% embryos affected, small and ectopic location of the thymus
**Mouse models of SCID and Nude/SCID:** ***Foxn1*** **mutant mice generated**				
*Foxn1 nu/nu*	Aplastic thymus	YES	DN1^a^	Required for TEC development, differentiation Regulates epithelial cells in the skin and nail beds
*Foxn1*ΔExon3	Hypoplastic thymus	NO	DN1^a^	Required for TECs Normal hair and nail beds
i*Foxn1*Δ7,8	Hypoplastic thymus	NO	DN2-DN3^a^	Inducible deletion of Foxn1 causes a loss of thymic structure, reduced T cell output
*Foxn1*^933/1089^	Severe hypoplasia of the thymus	NO	DN1^a^	Required for TEC development, differentiation. Normal hair and nail beds
*Foxn1*^1089/1089^	Hypoplastic thymus	NO	DP^a^	5 amino acid region required for DP^a^ to SP^a^. Normal hair and nail beds
**Mouse models of Otofaciocervical syndrome:** ***Pax1*** **mutations**				
*Undulated*	Mild hypoplasia	NO	None	Gly to Ser mutation causes reduced DNA binding activity. Affects the patterning of the thymic anlage
*Undulated short*	Mild hypoplasia	NO	None	125 kb region is deleted, including *Pax1* coding region. Affects the expression of a long non-coding RNA, which is lost while Nkx2.2 is increased
*Undulated extensive*	Mild hypoplasia	NO	None	Last exon of *Pax1* deleted. Affects the patterning of the thymic anlage
*Pax1*	Mild hypoplasia	NO	None	Complete *Pax1* knockout. Affects the patterning of the thymic anlage

a *Developmental stages of thymopoiesis: DN subset is CD4^−^CD8^−^; DN1, CD44^+^CD25^−^; DN2, CD44^+^CD25^+^; DN3, CD44^−^CD25^+^; DP, CD4^+^CD8^+^ thymocyte subset; SP, CD4^+^ CD8^−^ and CD4^−^CD8^+^ single positive subsets*.

b *N-ethyl-N-nitrosourea = ENU*.

In the mouse models, haploinsufficiency of *Tbx1* is generally not very penetrant in eliciting hypoplasia/aplasia of the thymus (90, 91, 94, 96, 100). By comparing embryos expressing varying levels of *Tbx1*, expression of this transcription factor at or below 35% normal values results in a more frequent and damaging thymic hypoplasia (101). Thymic hypoplasia resulting from the reduced levels of *Tbx1* are likely caused by developmental abnormalities in the pharyngeal region. However, the studies published to date have not concentrated on the 3rd PP. What is noticeably different are the 4th pharyngeal arches (PA), adjacent to the 3rd PPs, which are absent or developmentally delayed between day e9.5-11.5 of embryogenesis (Figure 1B) (101, 102). This impacts the patterning of the structures originating from the right and left 4^th^ PA, causing a displaced right subclavian artery and interrupted aortic arch type B, respectively. Both cardiac presentations are common clinical phenotypes of human 22q11.2del (102). *Tbx1* is specifically expressed in the regions comprising the pharyngeal arches as well as in the endothelial layer that juxtaposes the developing parathyroid (103). It is not expressed in the thymic anlage, suggesting that *Tbx1* haploinsufficiency does not directly impact TECs, consistent with the observations that enforced expression of *Tbx1* within the 3^rd^ PP actually represses TEC development (103). A plausible explanation for the thymic hypoplasia in 22q11.2del is that reduced levels of *Tbx1* in the pharyngeal region impact the neural crest-derived mesenchymal cells that surround the 3^rd^ PP. The importance of these mesenchymal cells and other cell types has been more clearly revealed in *Tbx1*-null embryos. An immunohistochemical analysis of these embryos reveals an abnormal distribution of proteins involved in the formation of the extracellular matrix, cell adhesions, and cell-cell contact (vinculin, paxillin and collagen) (104). Changes in the expression patterns of these proteins affects the NCC-derived mesenchyme along with the epithelial cells in the second heart field (104). Such results strongly suggest that the NCC-derived mesenchymal cells surrounding the 3^rd^ PP may also have abnormal mesenchymal and endothelial cell distributions required for the proper patterning of the 3^rd^ PP. This is likely what causes a size restriction on the developing thymus. In one mouse model of 22q11.2del (Tbx1^neo2/neo2^), the embryonic thymus is size restricted yet still supports normal T cell development (Table 2, Figure 1C). This indicates that the TECs are functional, matching the phenotype noted in the hypoplastic thymic lobes from 22q11.2del patients (83). In summary, mouse models of 22q11.2del strongly suggest that the initial developmental problems leading to thymic hypoplasia are coupled to mesenchymal cell defects. As the mesenchymal cells provide critical support functions for TECs, the consequence is reduced TEC expansion. Comparing 22q11.2del with other human clinical syndromes further supports this notion, as described next.

### Charge Syndrome Due to *Chromodomain Helicase DNA Binding Protein 7* Mutation

Coloboma-heart defects-atresia choanae-retardation of growth-genital abnormalities-ear abnormalities (CHARGE) is a multi-syndromic congenital disease (11, 105, 106). Approximately 90% of CHARGE patients have mutations in *Chromodomain Helicase DNA Binding Protein 7* (*CHD7*) (OMIM# 0214800) (107). CHD7 is an ATP-dependent nucleosome remodeling factor, regulating chromatin accessibility and consequently, gene expression (108). CHD7 also positively regulates ribosomal RNA biogenesis in the nucleolus (109). Affecting an estimated 1 in every 10,000 humans, 953 mutations have so far been discovered in *CHD7* (ClinVar database). These include missense, non-sense, deletion, splicing, and frame-shift mutations, resulting in a loss-of-function of varying severity depending on the location and/or effect of the mutation on the protein (105, 110). Patients with the CHARGE syndrome have immune system problems that contribute to their recurrent infections; otitis media, sinusitis, upper airway infections, pneumonia, and/or sepsis (106, 111). These infections are most often attributed to malformations of the craniofacial region, the upper respiratory tract, and the 7th cranial nerve (facial innervation). Of note, the first descriptions of CHARGE suggested that the infectious issues were of low incidence (105). More recent reports reveal that immune system complications are far more prevalent, with developmental problems of the thymus additionally reported as causal to the increased susceptibility to infections (106, 112). An immunological assessment of 59 CHARGE patients revealed that about 50% had a T^−/lo^B^+^NK^+^ phenotype (106). Immunoglobulin levels and subclasses were normal in most of these CHARGE patients. The absolute numbers of B cells, including memory cells, were very similar to that in controls. The low T cell numbers were a consequence of a thymic dysfunction as TREC levels for these patients were reduced relative to normal controls. Chart reviews for 36 CHARGE patients who had cardiac surgeries revealed 16 of 36 had a hypoplasia or aplasia of the thymus (106). The prevalence of the thymic hypoplasia may be higher in embryos, as a small/absent thymus was noted in seven of 10 CHARGE fetuses described in one study (113).

*Chd7* is required for the formation of the multipotent migratory neural crest cells that migrate throughout the body, establishing the bone, cartilage, peripheral nervous system, and cardiac structures (114). To understand the role of *Chd7* in CHARGE, especially given the varied congenital problems that can arise, various mouse models have been developed (115–118). The mouse models include those generated with gene-trapped ES cell lines, N-ethyl-N-nitrosourea (ENU) mutagenesis, targeted mutations in *Chd7*, and various floxed alleles of the gene (Table 2). Embryological analyses indicate that *Chd7* is expressed in the pharyngeal region, including the 3rd PP, the 4th PA, and the 1st PP, the latter forming the auditory tube and middle ear canal (119). As early as e10.5, the 4th PA is malformed or absent in 50% of the *Chd7* mutant embryos, resulting in an interrupted aortic arch type B and displaced right subclavian artery, just as with 22q11.2del (90). While most of the studies did not focus on thymus abnormalities, one group did report on this tissue. In this study, about 11% of e14.5 Chd7^+/xk^ embryos were found to have irregularly shaped thymic lobes, smaller and more oblong in appearance along with some ectopic positioning (119). *Chd7* is expressed in the surrounding mesenchyme and at higher levels in TECs, suggesting that the *Chd7* mutations impact both neural crest-derived mesenchymal cells and TECs (115). Interestingly, modulation of retinoic acid (RA) levels *in utero* can limit the severity of the phenotypes resulting from the *Chd7* mutations (120, 121). The clinical phenotypes due to retinoic acid embryopathies, including hypoplasia of the thymus, are discussed in a later section.

Complementing the murine models, Zebrafish studies have provided additional insights into how *Chd7* impacts thymic tissue specification. One technology commonly used in Zebrafish is a gene knockdown approach with morpholino oligonucleotides (MOs), creating morphants that have a block in transcript expression. *Chd7* morphants have a disrupted morphogenesis of the 3rd PP, with the migration and function of neural crest cells (NCCs) in this area impaired (122). Both bmp4 and bmp2 levels are significantly diminished in the chd7 morphants, again revealing the importance of these soluble proteins in establishing the thymus and parathyroid domains. At later developmental time points, the *Chd7* knockdown impairs the formation of the thymic capsule and vasculature. This is coupled with a reduced formation/expansion of the TECs that may involve impaired differentiation of the endothelial layer. Finally, the TECs have a substantial loss of *Foxn1* expression, providing a mechanistic basis for the hypoplasia due to TEC abnormalities (122). In summary, the *Chd7* knockdown impacts the NCC-derived mesenchymal cells along with the TECs, which suggests that CHARGE affects more stromal cell populations than 22q11.2del.

### Nude/SCID and SCID Phenotypes Linked to *FOXN1* Mutations

Autosomal recessive mutations in the *Forkhead Box N1* (*FOXN1*) transcription factor cause a T^−^B^+^NK^+^ SCID phenotype due to a thymic aplasia as well as alopecia universalis and nail plate dystrophy (OMIM #601705) (123–127). Three distinct autosomal recessive mutations in *FOXN1* have been reported for 10 patients to date, and these mutations result in a complete loss of protein function, impacting TECs and skin epithelial cells. Patients with compound heterozygous mutations in *FOXN1* have also been reported with an atypical phenotype, a thymic hypoplasia without the co-presenting alopecia and nail dystrophy (22). With the increasing number of infants noted to have low TRECs, the subsequent use of exome sequencing for them has uncovered many individuals with single allelic mutations in *FOXN1* (22, 128). While such affected individuals will likely recover normal T cell numbers as one allele remains functional, it is unclear what impact such single allelic mutations will have on T cell output later in life (129). To date, about 131 distinct mutations in human *FOXN1* have been reported, and while many are benign, there are >20 that have either complete or partial loss of function consequences (ClinVar database). The best clinical treatment option for patients with autosomal recessive or specific compound heterozygous mutations that contribute to a loss-of-function for *FOXN1* is a thymic tissue transplant (12). Yet, while bone marrow transplants have also been undertaken for such patients, the underlying defect lies with the TECs of the thymus (22). Paralleling the clinical findings with 22q11.2del, a thymic tissue transplant is the best option as this directly resolves the TEC anomalies.

In the thymus, *Foxn1* is the master transcriptional regulator of TEC development, supporting the differentiation of both cortical and medullary TEC subsets (45, 130–132). These TEC subsets are critical for establishing the repertoire of TCR-expressing T cells that are selected to recognize but not respond to self-peptide/MHC complexes (44, 45, 131). Foxn1 is a 648 amino acid long transcription factor that contains DNA binding and transactivation domains, both required for protein function (133, 134). The DNA binding domain of Foxn1 comprises three alpha helices, three beta sheets, and two loops (wings) (130, 135). The 3rd helix and the 2nd winged segment interact with the major and minor grooves of DNA, respectively (130, 135). The DNA binding sequence bound by *Foxn1* is GAa/cGC, present in about 500 target genes (132). The genes regulated by Foxn1 include keratins, keratin-associated proteins, cytokeratins, thymo-proteasome components, and cell surface proteins (132, 136). These proteins are important for both cortical and medullary TEC functions along with the extrusion of the hair shaft through the dermal layers of the skin and for nail bed formation (132, 137, 138). In many of the promoter/enhancer elements bound by Foxn1, there are CREB and Tp63 binding sites, suggesting cooperative gene regulation by multiple transcription factors (132).

Mouse and rat models have greatly aided in delineating the functions of *Foxn1*. First and foremost was a spontaneously arising mutant mouse line, discovered in 1966, with a pronounced nude phenotype (*nu/nu*). Almost three decades passed before the mapping of the *nu/nu* allele to autosomal recessive mutations in *Foxn1* (130, 134). The *nu* mutation results in a single base pair deletion in exon 3, causing a frameshift and almost no protein expression (130). The mice lack fur, whiskers, and nails (130, 139, 140). The thymus in the *nu/nu* mouse is a small cystic tissue that is unable to support TEC and consequently, thymocyte development (23, 141). Such nude mice are commonly referred to as Nude/SCID given their combined lack of fur and T^−^B^+^NK^+^ immune profile. An analysis of embryos from these mice show that *Foxn*1 is not required for the initial specification of the thymic region within the 3rd PP, but rather for the vascularization of this tissue along with TEC differentiation/expansion (142, 143). Nude rats (*rnuN, rnu*) and cats (*nu/nu*) with autosomal recessive mutations in *Foxn1* have similarly been described, with the first nude rat actually found in 1953, prior to the mouse reports (130, 144–146). While the *nu* and *rnuN* mutations prevent translation of the DNA binding and transactivation domains, much like the autosomal recessive *FOXN1* mutations in humans, *rnu* rats carry a mutation within exon 8, which creates a stop codon. This leads to the expression of a truncated protein (amino acids 1-473) lacking the transactivation domain. Characterizing this region revealed several aspartic acid residues essential for protein function (133). In an unrelated study, the introduction of a truncated *Foxn1* construct, wherein only exon 3 is deleted, blocks TEC development/expansion while allowing for hair extrusion and nail formation (147). It remains unclear how this occurs as both DNA binding and transactivation domains remain intact. In a separate cohort of mice developed to genocopy the compound heterozygous *FOXN1* mutations identified in an infant, the mice (Foxn1^933/1089^) had T^−^B^+^NK^+^ immune profile with normal hair growth and nail extensions (22). Unlike 22q11.2del and CHARGE, these *FOXN1* mutations directly impact TEC development, causing a loss of both DP and SP thymocytes (Figure 1C). One of the mutations in *FOXN1* (FOXN1^1089^) causes a loss of 5 amino acids at the very end of the DNA binding domain (p.W363C with a 5 amino acid loss). Knock-in mice harboring this mutation on both alleles (Foxn1^1089/1089^) have a selective block in thymopoiesis at the DP stage, with hair follicles and nails appearing normal (22). This 5-amino acid sequence is highly conserved with *Foxn4*, an ancestral ortholog of *Foxn1* (148). Interestingly, the cephalochordate species (lancelets) lack a thymus, and have a divergent sequence within this 5-amino acid stretch (22, 148). This suggests this small sequence is important for the expansion of DP thymocytes and their positive selection into CD4^+^ and CD8^+^ subsets (22). There is a 2nd patient described with distinct compound heterozygous mutations in *FOXN1* (FOXN1^1288/1465^). In functional assays, one of the mutations (Foxn1^1465^) leads to a p.R489fsX61 truncation of the protein, resulting in 18% normal transcriptional activity (22). This mutation prevents the translation of the transactivation domain, revealing a requirement for this region to maintain normal TEC functions. Of note, an increasing number of single allelic *FOXN1* mutations are being reported for patients initially presenting with low TRECs (22, 128). The subsequent characterization of these novel mutations will likely reveal the basis for the differential functions of *FOXN1* in TEC subsets vs. skin epithelial cells. Of note, one research group has identified a cis-regulatory element (RE) in the 1st intron of *Foxn1*, the targeting of which reduces TEC numbers and functions without any impact on skin epithelial cells (149). This RE is a target of the *Foxn1* DNA binding domain, revealing a positive autoregulatory loop (150). The possibility exists that human patients may contain such intronic *FOXN1* mutations, but these have not been reported to date as whole genome sequencing, which is not commonly done, would be required to uncover them.

Post-natally, *Foxn1* needs to be continuously expressed in TECs to maintain normal T cell output from the thymus (132). Thus, the inducible deletion of *Foxn1* in adult mice reduces thymic cellularity, and impacts the expansion of the DN1-DN4 subsets of thymocytes (132). In “old” mice, *Foxn1* levels in the thymus are reduced significantly, which partly explains the tissue involution (151–153). Restoring *Foxn1* in the aged thymus significantly improves thymic cellularity and T cell output (152–155). Taken together, the numerous human reports regarding single allelic mutations in *FOXN1* and the diverse mouse models are beginning to reveal key regulatory features of this critical transcription factor needed for T cell output throughout life.

### Otofaciocervical Syndrome Type 2 (OTFCS2) and *PAX1* Mutations

Loss-of-function mutations in *PAX1* lead to skeletal defects along with thymic hypoplasia in patients, the latter contributing to the T^−/lo^B^+^NK^+^ phenotype (13, 15, 17). Four such patients received bone marrow transplants (prior to identification of the *PAX1* homozygous mutations) in an attempt to correct their SCID presentations. Notably, the bone marrow transplants were unable to restore T cell development [reviewed in (17)]. The T cells, characterized in the patients after their bone marrow transplants, were of donor origin and exhibited a memory phenotype. Such findings are consistent with the current knowledge that *PAX1* regulates the patterning of the pharyngeal region, thereby impacting the stromal cell populations that would not be corrected by a bone marrow transplant.

Using embryos isolated from pregnant mice, *Pax1* transcripts are evident in the four pharyngeal pouches as early as e10.5, and become confined to mesenchymal condensations as embryogenesis progresses (29). This transcription factor is present in the 3rd PP endoderm and is subsequently detected in a subset of TECs during embryogenesis (29). Its expression is retained in the adult thymus. The deletion of *Pax1* results in a marginal hypoplasia of the thymus (29, 30). This was reported in the *undulated* series of mouse lines that had varying mutations within *Pax1* or with surrounding regulatory elements. These mice were initially described in the 1940's due to their kinked tails and vertebral deformities (Table 2) (29, 156). All *undulated* mutants have a smaller thymus about 2/3rd normal size (29, 30). Interestingly, only in the context of a *Hoxa3* haploinsufficiency does the thymus in the *Pax1* mutant lines exhibit a more severe hypoplasia, with the two lobes ectopically positioned (30). The mild thymus phenotypes in the mouse models comprising various *Pax1* mutations sharply contrast the severe hypoplasia in humans with *PAX1* autosomal recessive mutations. One possible explanation is a compensatory contribution by murine *Pax9* when *Pax1* is lacking. *Pax9* overlaps in expression with *Pax1* in the endodermal-derived epithelium of the pharyngeal pouches (157). In mice, a complete deficiency of *Pax9* causes a thymic aplasia and a lack of teeth, while in humans, autosomal recessive mutations in *PAX9* cause selective tooth agenesis (158, 159).

### Clinical Conditions During Pregnancy Leading to a Thymic Hypoplasia/Aplasia

Maternal diabetes and systemic use of retinoic acid (RA) derivatives during pregnancy can cause long-term thymic hypoplasia in newborns (18–21). What's more, gestational diabetes leads to congenital malformations in the developing fetus which overlap with those noted in individuals with 22q11.2del; hypoplasia/aplasia of the thymus, cardiac outflow tract defects, and hypoparathyroidism (160–163). Estimates suggest that 18% of infants who required a thymic tissue transplant due to an aplasia of this tissue, and did not have 22q11.2del, were born to mothers who had maternal diabetes (72). In spite of the obvious overlap in clinical presentations between 22q11.2del and diabetic embryopathy, it remains unknown how blood sugar dysregulation affects the pharyngeal apparatus.

In rodent studies, the induction of diabetes in pregnant mice and rats causes thymic hypoplasia along with intrauterine growth impairment (164). While intrauterine growth delay will certainly contribute to thymic hypoplasia, there is some evidence that the hypoplasia can result from patterning defects within the pharyngeal apparatus. As gestational diabetes in rodent models is difficult to regulate, the use of a pregestational diabetes mouse model has revealed that retinoic acid production is dysregulated in the developing embryos. Thus, pregestational diabetes reduces the expression of *Cytochrome P450 family 26 subfamily A member 1* (*Cyp26a1*), an enzyme that catabolizes retinoic acid (RA) in the caudal region (tailbud) of developing embryos (165). RA is a derivative of Vitamin A, which functions as a natural morphogen regulating the patterning of the 3rd PP along with the 4th PA (166–170). Both reductions and elevations in RA can lead to hypoplasia of the thymus along with the other congenital malformations that overlap remarkably with 22q11.2del and CHARGE phenotypes (8). While the levels of Cyb26a1 or related family members within the pharyngeal region were not assessed in the pregestational diabetes model, their loses would increase RA, which could cause the problems of the thymus. Consistent with this, injecting high levels of RA in pregnant mice at e9.5 results in the formation of a hypoplastic/aplastic thymus, examined at e11.5–e12.5 (21). It is known that high levels of RA can impair the migration of the NCCs in the region surrounding the 3rd PP (21). Moreover, high levels of RA can reduce the expression of *Pax1* within the 3rd PP and *Tbx1* throughout the pharyngeal apparatus (171–173). RA likewise represses Bmp4 activity, impacting thymic tissue specification and development by ultimately reducing the levels of *Foxn1* (174). These changes have some similarity to that described for embryos developing in the setting of *Chd7* mutations.

The second medical condition that can lead to permanent hypo- or aplasia of the thymus in newborns is exposure to elevated levels of RA during pregnancy. Drugs such as tretinoin or isotretinoin are retinoids prescribed to patients to both reduce the severity of their acne and smoothen the skin. However, if taken during pregnancy, the higher levels of RA can trigger 22q11.2-like congenital malformations in the developing embryos (18, 72, 160–162, 175, 176). The mechanism for this hypoplasia is a combination of *Tbx1, Pax1*, and *Foxn1* suppression, as described in the preceding sections of this review (Figure 1A). The profound damage caused by RA has led to a generalized warning from the FDA for women to avoid treatments with RA derivatives during pregnancy.

## Conclusion

A number of clinical conditions impact the specification of the thymus during embryogenesis. Interestingly, those that affect the stromal cell populations have overlapping phenotypes, revealing that many of the affected genes function in related developmental pathways. 22q11.2del appears to impact one of the earlier stromal cell types involved in this process, the NCC-derived mesenchymal cells. These cells regulate the patterning and formation of the thymic anlage. CHARGE affects mesenchymal cells, endothelial cells and the TECs, while *FOXN1* mutations selectively affect the TECs. It is becoming obvious that the three stromal cell types have considerable cross-talk to coordinate the formation and expansion of the thymus. A balanced interplay among all three is essential for the normal specification and expansion of the thymic tissue. Variations in the functions of any one of these stromal cell populations will impact the other, which likely explains the overlapping clinical phenotypes noted among affected individuals.

## Ethics Statement

Animal work described in this article has been approved and conducted under the oversight of the UT Southwestern Institutional Animal Care and Use Committee (APN numbers: 2015-101163 and 2015-101247).

## Author Contributions

PB and NO generated the figures. PB, CW, and NO wrote and modified the manuscript.

## Conflict of Interest

The authors declare that the research was conducted in the absence of any commercial or financial relationships that could be construed as a potential conflict of interest.
